# NusG, an Ancient Yet Rapidly Evolving Transcription Factor

**DOI:** 10.3389/fmicb.2020.619618

**Published:** 2021-01-08

**Authors:** Bing Wang, Irina Artsimovitch

**Affiliations:** Department of Microbiology and the Center for RNA Biology, The Ohio State University, Columbus, OH, United States

**Keywords:** antitermination, evolution, NusG, RfaH, transcriptional pausing, termination, virulence

## Abstract

Timely and accurate RNA synthesis depends on accessory proteins that instruct RNA polymerase (RNAP) where and when to start and stop transcription. Among thousands of transcription factors, NusG/Spt5 stand out as the only universally conserved family of regulators. These proteins interact with RNAP to promote uninterrupted RNA synthesis and with diverse cellular partners to couple transcription to RNA processing, modification or translation, or to trigger premature termination of aberrant transcription. NusG homologs are present in all cells that utilize bacterial-type RNAP, from endosymbionts to plants, underscoring their ancient and essential function. Yet, in stark contrast to other core RNAP components, NusG family is actively evolving: horizontal gene transfer and sub-functionalization drive emergence of NusG paralogs, such as bacterial LoaP, RfaH, and UpxY. These specialized regulators activate a few (or just one) operons required for expression of antibiotics, capsules, secretion systems, toxins, and other niche-specific macromolecules. Despite their common origin and binding site on the RNAP, NusG homologs differ in their target selection, interacting partners and effects on RNA synthesis. Even among housekeeping NusGs from diverse bacteria, some factors promote pause-free transcription while others slow the RNAP down. Here, we discuss structure, function, and evolution of NusG proteins, focusing on unique mechanisms that determine their effects on gene expression and enable bacterial adaptation to diverse ecological niches.

## Introduction

In every living cell, multi-subunit RNA polymerases (RNAPs) carry out the first step of gene expression, transcription of a DNA template into an RNA copy. Reflecting their common evolutionary origin in the last universal common ancestor (LUCA) and the basic mechanism of RNA synthesis, RNAPs share an overall architecture and structural elements that play key roles in the assembly of transcription complexes, substrate selection and catalysis, interactions with nucleic acids, etc. (Lane and Darst, 2010a,b). However, extant RNAPs differ greatly in subunit composition and sequence: core RNAPs are composed of 5–7 subunits in bacteria vs. 12+ subunits in archaea and eukaryotes, and even RNAPs from mesophilic bacteria *Escherichia coli* and *Bacillus subtilis* are only 50% identical. Differences in cellular transcriptional machinery are thought to reflect unique regulatory constraints imposed by diverse habitats. In support of this notion, even basal general transcription factors that assist RNAP during each step of the transcription cycle are not conserved between kingdoms. The sole exception to this trend is a transcription elongation factor NusG (Werner, 2012).

Bacterial Nus (N-utilization substance) proteins have been identified genetically based on their requirement for the coliphage λ development (Casjens and Hendrix, 2015). In *E. coli* and *Salmonella*, potentially harmful xenogenes are silenced by premature transcription termination by a hexameric RNA helicase Rho (Peters et al., 2012; Bossi et al., 2019). To escape silencing, bacteriophages have evolved antitermination mechanisms targeting Rho or RNAP (Santangelo and Artsimovitch, 2011). The immediate early gene *N* of phage λ is required for the expression of delayed-early genes. N nucleates the assembly of a large transcription antitermination complex (TAC) composed of RNAP and NusABEG proteins (Mason and Greenblatt, 1991; Krupp et al., 2019) and a similar TAC assembles during transcription of the *E. coli* ribosomal RNA operons (Squires et al., 1993; Huang et al., 2020). NusA and NusG are general transcription elongation factors, which are associated with RNAP transcribing all genes, at least in *E. coli* (Mooney et al., 2009a). NusE, *a.k.a.* the ribosomal protein S10, requires a binding partner NusB to remain soluble while not a part of the ribosome; NusB is selectively enriched on rRNA operons (Mooney et al., 2009a), consistent with its principal role in rRNA synthesis. Among the shared components of the TACs, NusG is the only factor that facilitates transcription elongation *in vivo* and *in vitro* (Burova et al., 1995; Burns et al., 1998; Zellars and Squires, 1999); by contrast, NusA increases RNAP pausing and intrinsic termination, whereas NusB/E have no effect (Belogurov and Artsimovitch, 2015).

All NusG-like proteins (NusG in bacteria; Spt5 in archaea and yeast, DSIF in mammals) bind to an evolutionary conserved site on the largest RNAP subunit (Klein et al., 2011; Martinez-Rucobo et al., 2011; Ehara et al., 2017; Kang et al., 2018; Vos et al., 2018). The NusG binding site is located on the tip of the RNAP clamp, a conserved flexible module that closes over the DNA binding channel. The clamp closes during the formation of a transcriptionally competent initiation complex, remains closed throughout elongation, and opens during termination (Belogurov and Artsimovitch, 2019); more subtle movements of the clamp have been proposed to accompany RNAP pausing, which serves as a prelude to termination (Kang et al., 2019). By keeping the clamp locked, NusG proteins are thought to promote continuous, pause-free RNA synthesis, an essential function given that the premature release of the RNA transcript is irreversible. The presence of a clamping factor in LUCA thus underscores the fundamental importance of transcription processivity, particularly on difficult templates (Werner, 2012).

The antipausing and, by inference, antitermination activity of NusG prompted its annotation as a transcription antiterminator. Likewise, many subsequently discovered bacterial NusG homologs have been shown to possess antitermination activity (Artsimovitch and Knauer, 2019). Nevertheless, this view has been challenged since the time of *E. coli* NusG discovery by the data in support of its role as a termination-promoting factor. NusG is essential in wild-type *E. coli* (Downing et al., 1990) and its depletion leads to defects in Rho-dependent termination (Sullivan and Gottesman, 1992). NusG aids Rho in silencing transcription of damaged and harmful RNAs genome-wide (Peters et al., 2012) and promotes efficient termination by Rho *in vitro* (Burns and Richardson, 1995). Indeed, the *nusG* gene can be deleted, albeit at a significant fitness cost, in an *E. coli* strain lacking the toxic *rac* prophage, which is silenced by Rho (Cardinale et al., 2008). Point mutations in *nusG* that lead to defects in transcription termination (Saxena and Gowrishankar, 2011) or interactions with the ribosome (Saxena et al., 2018) do not have significant fitness phenotypes.

**FIGURE 1:**
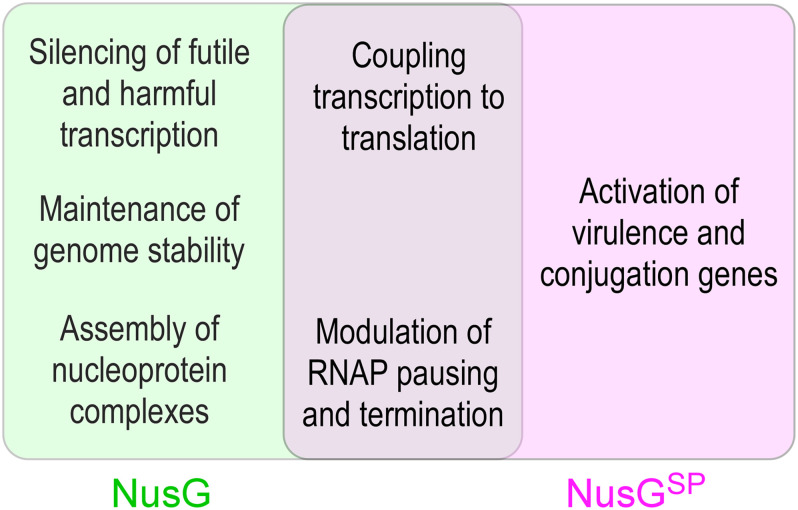
Unique and overlapping cellular functions of housekeeping *E. coli* NusG and its specialized paralogs.

Functional studies of NusG-like proteins from different bacteria support a picture in which these factors can mediate diverse effects on RNA synthesis (Figure 1). Through contacts to RNAP, nucleic acids, and auxiliary proteins, NusG homologs can suppress or promote transcriptional pausing and termination and bridge RNAP to other cellular machineries. Most unusually for a family of alternative transcription regulators, although binding to the same site on the transcribing RNAP, NusG-like proteins frequently have exactly opposite effects on the expression of some genes, most notably those encoding virulence determinants. Furthermore, even the housekeeping NusG proteins have seemingly opposite effects on RNA synthesis; for example, unlike its *E. coli* counterpart, *B. subtilis* NusG promotes RNAP pausing *in vitro* and *in vivo* (Yakhnin et al., 2016, 2020a). Below, we describe recent advances in our understanding of molecular mechanisms, evolution, and regulatory diversity of bacterial NusG-like proteins.

## Structure and Target Conservation

**FIGURE 2:**
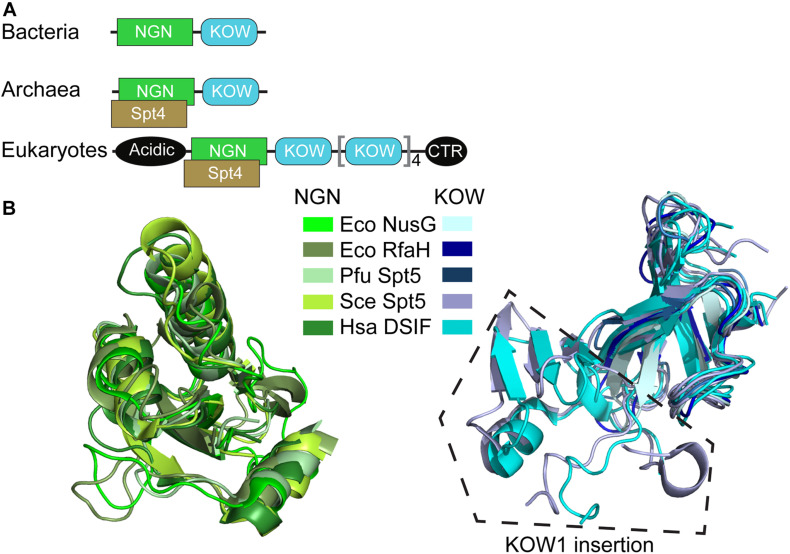
Structural conservation of NusG-like proteins. **(A)** Domain organization. **(B)** Superposition of NGN and KOW domains. PDB IDs: *E. coli* (Eco) NusG-NGN: 2K06; Eco NusG-KOW: 2KVQ; Eco RfaH-NGN: 2OUG; Eco RfaH-KOW: 2LCL; *Pyrococcus furiosus* (Pfu) Spt5-NGN/KOW: 3P8B; *Saccharomyces cerevisiae* (Sce) Spt5-NGN: 2EXU; Sce Spt5-KOW1: 4YTK; Sce Spt5-KOW2/3: 4YTL; *Homo sapiens* (Hsa) DSIF-NGN: 3H7H; Hsa DSIF-KOW1: 5OIK; Hsa DSIF-KOW2: 2E6Z; Hsa DSIF-KOW3: 2DO3; Hsa DSIF-KOW4: 5OHO; Hsa DSIF-KOW5: 2E70. Sce Spt5-KOW1/2/3 have similar structures and are shown in the same color, as are Hsa DSIF-KOW1/2/3/4/5 domains.

NusG-like proteins have a similar structural core consisting of a NusG N-terminal domain (NGN) and a C-terminal domain with a 27-residue long Kyrpides-Ouzounis-Woese (KOW) motif common among RNA-binding proteins (Kyrpides et al., 1996; Ponting, 2002; Figure 2). Bacterial NusG alone can perform its function, while Spt5 has an obligatory partner—a small zinc finger protein Spt4 (called RpoE in archaea). Eukaryotic Spt5 contains several KOW domains, the first of which carries a large insertion, an N-terminal acidic region, and an unstructured C-terminal repeat (CTR) domain (Figure 2A); in metazoan DSIF, additional KOWs are present at the very C terminus of the protein (Decker, 2020). Apart from the KOW1 insertion, the NGN and KOW domains from all life have very similar topologies (Figure 2B).

All NGN domains make very similar contacts to two conserved RNAP elements (Klein et al., 2011; Martinez-Rucobo et al., 2011; Ehara et al., 2017; Kang et al., 2018), the clamp helices (CH) in the largest RNAP subunit (β’ in Bacteria) and the gate loop in the second largest subunit (β in Bacteria).

In addition, some NGNs make sequence-specific contacts to the non-template DNA strand in the transcription bubble of the transcription elongation complex (TEC; see below). The NGN binding site on the TEC is structurally analogous to binding sites of transcription initiation factors in promoter complexes; e.g., bacterial σ factors recognize non-template DNA sequences and an adjacent region on the β’ CH during promoter-dependent initiation (Zhang et al., 2012). Consequently, NusG/Spt5 proteins compete with the cognate initiation factors for binding to RNAP, reducing pausing during transcription elongation and potentially facilitating promoter escape (Sevostyanova et al., 2008; Grohmann et al., 2011). Along with the housekeeping NusG present in every free-living cell, many species also contain NusG paralogs (Wang B. et al., 2020) that regulate expression of selected genes in a sequence- or condition-specific fashion.

While the “clamping” contacts between the NGN and TEC are sufficient for NusG/Spt5 effects on RNA synthesis (Mooney et al., 2009b; Hirtreiter et al., 2010), the KOW domains determine their regulatory properties. In *E. coli* NusG, interactions between the KOW domain and Rho facilitate termination (Lawson et al., 2018), whereas the KOW-ribosome interactions couple transcription to translation (Saxena et al., 2018). In eukaryotic Spt5, the presence of multiple KOWs and the CTR, which acts as a hub for recruitment of several RNA processing enzymes and other cellular factors (Decker, 2020), expands the range of regulatory interactions.

## Silencing Aberrant Transcription

Accurate and timely execution of the gene expression program is essential for cell survival. By itself, RNAP is a passive interpreter of genetic information. Auxiliary proteins instruct RNAP to synthesize RNAs that are required for proper cellular function and prevent it from wasting resources on making useless or potentially harmful RNAs, such as antisense transcripts or mRNAs encoding toxic proteins. In *E. coli*, the housekeeping NusG travels with RNAP transcribing almost all genes (Mooney et al., 2009a), save a few controlled by its paralog RfaH (Belogurov et al., 2009), actively contributing to the transcriptome surveillance. *First*, NusG cooperates with Rho to silence transcription of aberrant RNAs; this is an essential function of *E. coli* NusG (Mitra et al., 2017). *Second*, NusG increases RNAP processivity by modifying properties of the TEC, a shared function of NusG proteins from all life. *Third*, NusG is an integral part of multi-component nucleoprotein complexes that promote facile synthesis and proper assembly of the ribosomal RNAs, and thus the ribosomes. *Finally*, NusG helps to protect translatable mRNAs from premature release by Rho by bridging the RNAP and the ribosome.

### Rho-Dependent Termination

Rho is an ATP-dependent, RecA-type hexameric helicase that terminates transcription of a wide variety of genes in bacteria. Initially viewed as a sequence-specific terminator that requires a C-rich Rho utilization (*rut*) element for loading onto the nascent RNA and subsequent TEC dissociation, Rho has recently emerged as a global multi-functional regulator (Mitra et al., 2017). In addition to its canonical role, inducing termination at the end of some genes (Peters et al., 2012), Rho silences transcriptional noise and expression of horizontally acquired genes, reduces translational stress, and prevents replication-transcription collisions. Genome-wide studies demonstrate that *E. coli* Rho travels with the elongating RNAP, together with NusG and NusA (Mooney et al., 2009a), from the onset of elongation, and acts on numerous cellular targets that lack easily recognizable *rut* sequences (Peters et al., 2012).

**FIGURE 3:**
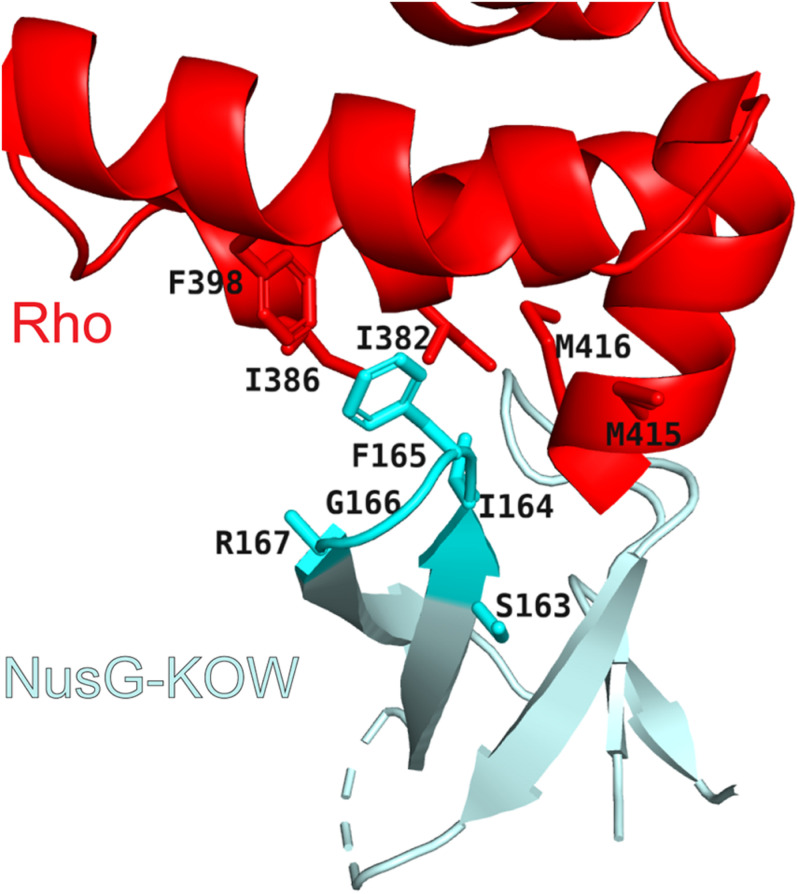
Rho/NusG-KOW interface. Rho residues that contact NusG are shown as red sticks. NusG KOW residues implicated in Rho and S10 binding are shown as cyan sticks; PDB ID: 6DUQ.

To silence AT-rich xenogenes and trigger the release of antisense transcripts or low-quality mRNAs independently of their sequence, Rho relies on help from NusG, which has been implicated in Rho termination at suboptimal, C-less sites (Peters et al., 2012). In a binary system lacking RNAP, NusG activates Rho by promoting isomerization from an open-ring, RNA-loading state, to a closed-ring, translocation-competent state, the transition otherwise triggered by a perfect *rut* element in the RNA (Lawson et al., 2018). The NusG KOW interacts with the C-terminal translocase domain of Rho (Figure 3), inducing conformational changes that favor the ring closure even on RNAs devoid of C residues (Lawson et al., 2018). NusG-Rho contacts are mediated by the same KOW region that binds to the ribosomal protein S10 (Burmann et al., 2010), explaining why the translating pioneering ribosome protects the mRNA from a spurious attack by Rho. By contrast, the corresponding Rho-binding residues are missing in RfaH (Lawson et al., 2018), explaining why RfaH does not bind to Rho.

**FIGURE 4:**
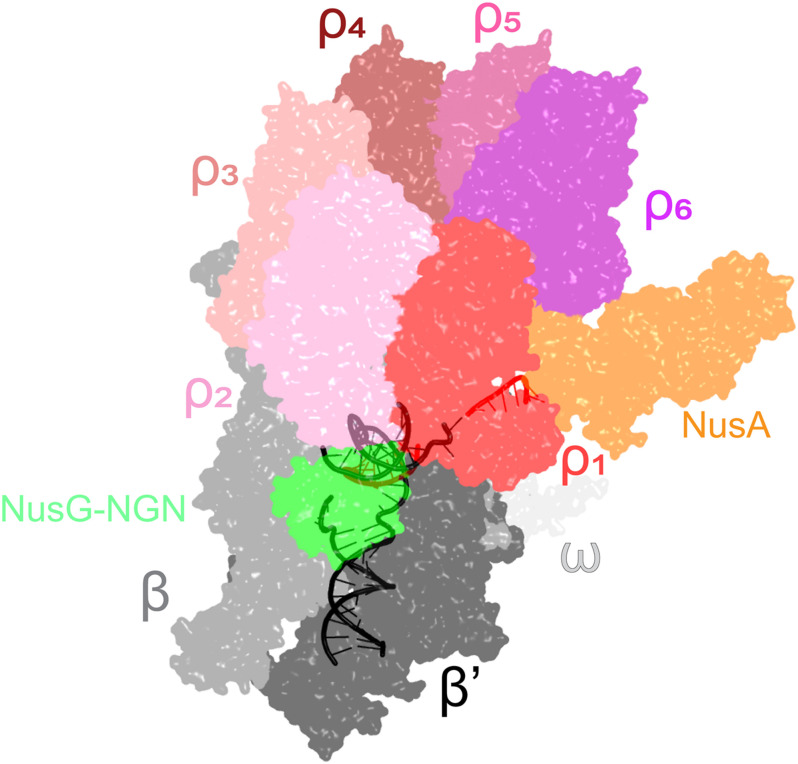
A cryo-EM structure of the Rho engagement complex, in which Rho hexamer makes initial contacts with the transcribing RNAP bound to NusA and NusG. The DNA is shown in black; the RNA—in red. PDB ID: 6Z9P.

However, the ring closure activity of NusG may not be the main mechanism by which NusG stimulates Rho-dependent termination. Consistent with biochemical data (Schmidt and Chamberlin, 1984; Epshtein et al., 2010) and genome-wide mapping (Mooney et al., 2009a) that support persistent Rho-RNAP interactions, a recent cryo-EM analysis of the *E. coli* TEC under attack by Rho reveals seven complexes thought to represent sequential steps in the termination pathway (Said et al., 2020). During the initial binding to the TEC, Rho makes numerous contacts to the RNAP subunits, NusA and NusG NGN (Figure 4), but captures the nascent RNA transcript only later in the pathway. Once engaged, Rho induces dramatic conformational changes in RNAP and Nus factors, which ultimately trap a moribund TEC in which the clamp is wide open and the RNA 3′ end is dislodged from the RNAP active site (Said et al., 2020), a model initially proposed by Nudler and colleagues (Epshtein et al., 2010). In this structurally defined pathway, NusG NGN assists Rho loading onto the RNA and then dissociates to allow for Rho-mediated RNAP clamp opening, whereas NusG KOW is invisible. Remarkably, the Rho ring remains opens even in the moribund TEC, implying that the NusG-promoted Rho helicase activity is required to unwind the RNA:DNA hybrid only after RNAP inactivation; this model is supported by a report that the *E. coli rho* gene becomes dispensable in the presence of a heterologous RNA:DNA helicase (Leela et al., 2013). The allosteric model of termination explains how Rho selectively binds to RNAs that are still being made and reinforces the notion that, even in bacteria, transcriptional regulators act in the context of multi-protein complexes, rather than on RNAP alone.

Indeed, recent evidence suggests that Rho and NusG cooperate with the histone-like nucleoid-structuring (H-NS) protein, a prototypical xenogeneic silencer, to limit unwanted gene expression. In *E. coli*, Rho and H-NS co-localize on the chromosome (Chandraprakash and Seshasayee, 2014) and mutations in *rho* and *hns* lead to synergistic growth defects (Peters et al., 2012). In *Salmonella*, depletion of NusG leads to massive upregulation of H-NS silenced loci, which include pathogenicity islands and are devoid of *rut* sites; consistently, mutations that compromise Rho-*rut* contacts have no effect on NusG-mediated silencing (Bossi et al., 2019). While the molecular mechanism of this cooperation remains to be determined, it likely reflects RNAP stalling when running into nucleoprotein filaments assembled by H-NS and other nucleoid-associated proteins on the template DNA (Boudreau et al., 2018).

### Inhibition of RNAP Pausing

**FIGURE 5:**
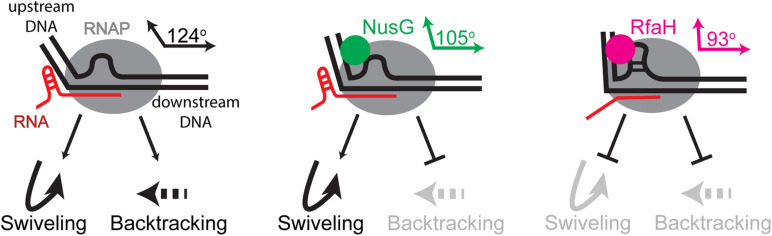
Antipausing activities of *E. coli* NusG and RfaH. Upon encountering a pause-inducing sequence, RNAP can either backtrack or undergo conformational changes termed swiveling; the latter are stabilized by formation of a pause hairpin in the nascent RNA. The NGN domains of both proteins bind near the upstream edge of the transcription bubble, promoting forward and thus inhibiting backward translocation. Transient (NusG) or stable (RfaH) interactions with the non-template DNA strand bring the upstream and downstream DNA duplexes closer together (indicated by angles between these duplexes), an effect that is more pronounced with RfaH. RfaH also binds to the β’ and β subunits with higher affinity, restricting the clamp movements to inhibit swiveling and hairpin-stabilized pausing. NusG lacks this activity.

During transcription of cellular DNA, RNAP frequently encounters unfavorable sequences or obstacles, such as DNA-bound proteins or DNA lesions, that slow the enzyme down or induce arrest. Retrograde movement of the RNAP along the RNA and DNA chains, or backtracking, is a common mechanism of pausing and arrest (Nudler, 2012). Backtracked complexes are rendered inactive because the nascent RNA is extruded through the active site, blocking nucleotide addition (Figure 5). The arrested complexes are long-lived, blocking progression of other RNAPs and replisomes, and must be released or reactivated upon transcript cleavage. Cleavage of the backtracked RNA, which is mediated by the RNAP active site and is strongly enhanced by Gre cleavage factors (Sosunova et al., 2003), repositions the 3’ end of the RNA in the active site. By preventing backtracking, an activity well-documented in the case of NusG and RfaH (Svetlov et al., 2007; Herbert et al., 2010), NusG-like proteins facilitate processive transcription and promote genome stability. Recent functional and structural data suggest a molecular mechanism of enhanced RNAP processivity, in which the NGN domain loops out the non-template DNA, bringing the upstream and downstream DNA duplexes closer together (Turtola and Belogurov, 2016; Kang et al., 2018; Nedialkov et al., 2018), and establishes contacts to the upstream DNA duplex (Krupp et al., 2019; Said et al., 2020). Together, these interactions alter the upstream DNA trajectory (Figure 5) and stabilize the upstream edge of the transcription bubble, which must melt to allow backtracking, explaining how NusG and RfaH inhibit backtracking (Svetlov et al., 2007; Herbert et al., 2010). In addition, the NGN domain, at least in the case of RfaH (Kang et al., 2018), disfavors subtle conformational changes (termed swiveling) that accompany the formation of hairpin-stabilized paused TEC (Kang et al., 2019) and constrains the path of the non-template DNA, preventing it from assuming non-productive conformations (Nedialkov et al., 2018); a similar mechanism has been proposed for yeast Spt5 (Crickard et al., 2016). Together, the NGN-promoted changes in the TEC ensure pause-free RNA synthesis, preventing arrest and termination.

### NusG-Assisted Antitermination

To enact RNA surveillance, Rho travels with the elongating RNAP and probes the nascent RNA “translatability.” RNAs that contain premature stop codons or are poorly translated, e.g., under conditions of proteotoxic stress, are released by Rho (Richardson, 1991). Yet a very large fraction of cellular RNA is never translated, most notably the most abundant and absolutely essential rRNA which comprises ∼50% of the newly synthesized RNA during the exponential growth phase (Dennis et al., 2004). Thus, making rRNA rapidly while protecting it from Rho is key to the survival of cells. Similarly, phage replication is critically dependent on uninterrupted transcription of the phage genome, but Rho is known to broadly silence xenogenes, including phages (Mitra et al., 2017).

**FIGURE 6:**
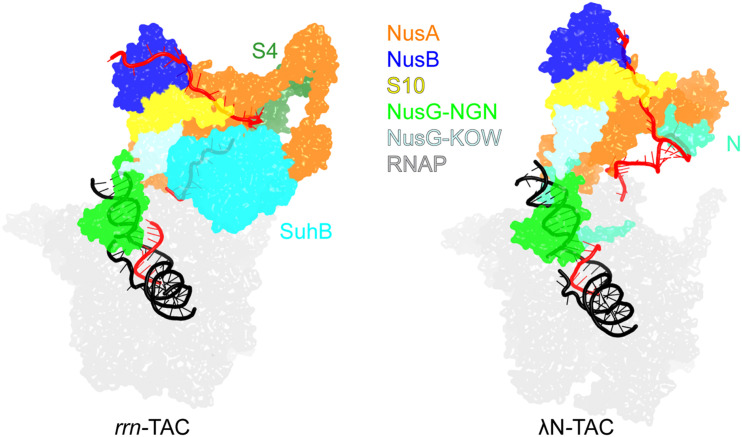
Transcription antitermination complexes (TAC). Left, *rrn*-TAC; right, phage λN-TAC. RNA is in red, DNA is in black. The unique proteins that play key roles in antitermination are shown on each complex; the shared components are indicated in the middle. PDB ID: *rrn*-TAC, 6TQO; λN-TAC, 6GOV.

Protection of the phage λ early genes and *E. coli* rRNA operons (*rrn*) from Rho is conferred by multicomponent TACs. Recently solved cryo-EM structures of these TACs (Figure 6) revealed common and unique details of their action (Krupp et al., 2019; Huang et al., 2020). Both complexes assemble on *boxA* and *boxB* elements in the nascent RNA and share a set of NusABEG factors. Each complex also includes unique factors, N in the λN-TAC and an inositol monophosphatase SuhB dimer + the ribosomal protein S4 in the *rrn*-TAC.

The λN-TAC is resistant to pausing and termination elicited by hairpin signals and Rho. An intrinsically unstructured λN is the principal player which uses a range of mechanisms to modify the TEC (Krupp et al., 2019). λN snakes inside the RNAP, making contacts to multiple RNAP domains and repositioning others, and rearranges Nus factor interactions. λN stabilizes the elongation-competent state of RNAP, inhibiting the nascent RNA hairpin formation and its stabilization by NusA, supports the anti-backtracking and anti-swiveling action of the NusG NGN domain. In the λN-TAC, neither NusG domain can make contacts to Rho observed in the binary Rho-NusG complex (Lawson et al., 2018) and Rho-TEC (Said et al., 2020) structures. Consequently, in the λN-TAC, NusG anti-pausing activity is augmented while its termination-promoting activity is abolished.

Although the *rrn*-TAC has a different protein composition, analogous structural changes inhibit backtracking and NusA-stabilized hairpin pausing and sequester NusG from Rho, with a much larger, well-folded SuhB dimer playing a central role in restructuring of the TAC components instead of λN (Singh et al., 2016). Notably, in addition to promoting pause- and termination-free RNA synthesis, the *rrn*-TAC acts as a molecular chaperone that actively assists the folding and maturation of the nascent RNA (Huang et al., 2020). Similarly to the ribosome-associated chaperones, SuhB, S4 and Nus factors assemble into a ring around the RNA exit channel, extending the channel outward to accommodate a longer segment of the exiting RNA. The RNA is thus sequestered away from the upstream DNA, blocking formation of deleterious R-loops, and is held within a positively charged protein cage to promote folding of local secondary structures and annealing of distant segments, which is required for processing of rRNA precursors into mature forms (Young and Steitz, 1978).

NusG plays a supporting role in both TACs: e.g., λN alone has a short-range antitermination activity and requires the TAC assembly to act over long distances (Rees et al., 1996). By contrast, RfaH is a principal, self-sufficient antiterminator: RfaH acts over very long distances yet its activity is not affected by cellular factors, at least *in vitro* (Artsimovitch and Landick, 2002). Other NusG^*SP*^ may similarly act alone.

### Transcription-Translation Coupling

In prokaryotic cells, the lack of a nuclear membrane provides an opportunity for direct physical interaction of the transcribing RNAP and the translating ribosome. The translation-coupled synthesis of the nascent mRNA is known as transcription-translation coupling. The coupling was directly observed by electron microscopy in 1970 in *E. coli* cells (Miller et al., 1970) and subsequently in archaeon *Thermococcus kodakarensis* (French et al., 2007). RNAP and ribosomes form a one-to-one complex with about 1 μM dissociation constant, which is already well within a physiologically relevant range, even in the absence of the nascent mRNA and accessory factors (Fan et al., 2017), resulting in factor-free coupling. Alternatively, the two complexes can be linked by bridging factors, e.g., via the NusG:S10 captured by NMR (Burmann et al., 2010). Substitutions at the *E. coli* NusG:S10 binding interface weakened NusG:S10 association *in vivo* and completely abolished it *in vitro* (Saxena et al., 2018).

**FIGURE 7:**
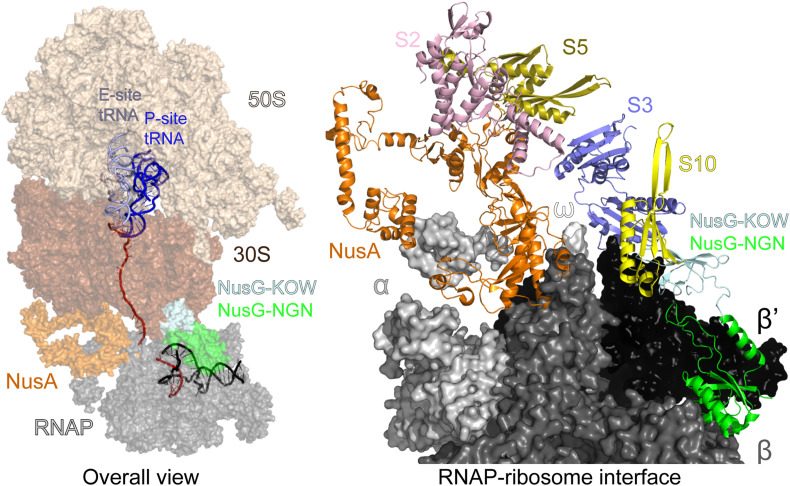
Transcription-translation coupling. Left—an overall view: mRNA is in red, DNA is in black. The interface between the ribosomal 30S subunit and RNAP is stabilized by NusA and NusG. Right—a view of the coupling interface; mRNA, DNA, the entire 50S and most of the 30S subunit have been removed for clarity. PDB ID: 6 × 7F.

The TEC-ribosome complexes, stabilized by general transcription factors, have been observed *in vitro* using cryo-EM (Wang C. et al., 2020; Webster et al., 2020) and analyzed inside cells using a combination of cross-linking mass spectrometry and cryo–electron tomography (O’Reilly et al., 2020). Evidence suggests that coupling may occur initially via direct RNAP:ribosome contacts and then is aided by accessory factors (Washburn et al., 2020). In the NusG/NusA coupled complex, the RNAP β’ subunit contacts the 30S subunit protein S3, NusA simultaneously binds to α/β subunits and S2/S5, and finally NusG binds to β/β’ and S10 (Figure 7). If the ribosome approaches the RNAP further, the collided state, in which the ribosome translocation and the factor-mediated coupling are no longer possible, forms (Wang C. et al., 2020; Webster et al., 2020). Preventing such unproductive collisions may be another function of NusA and NusG.

Since RNAP might often transcribe without a linked ribosome (Chen and Fredrick, 2018), the coupling events must carry important regulatory information (McGary and Nudler, 2013). The closely coupled ribosome prevents the formation of R-loops and RNAP backtracking, thereby promoting genome stability (Gowrishankar and Harinarayanan, 2004; Proshkin et al., 2010; Stevenson-Jones et al., 2020) and inhibits factor-independent termination by blocking the formation of nascent RNA hairpins (Roland et al., 1988). The coupled ribosome also prevents mRNA degradation, by blocking the access of RNaseE (Iost and Dreyfus, 1995), or premature Rho termination, by sequestering NusG and shielding the nascent RNA (Washburn et al., 2020). When the coupling is broken, e.g., by the ribosome pausing or stalling, Rho releases the nascent RNA, a phenomenon known as polarity (Richardson, 1991). Transcription attenuation is another regulatory mechanism dependent on coupling between the RNAP and the trailing ribosome, wherein the formation of an RNA hairpin induces RNAP pausing and the trailing ribosome pushes the RNAP out of the pause (Turnbough, 2019). By stabilizing the RNAP-ribosome tandem or aiding Rho, NusG controls the fate of the nascent RNA, promoting its translation or release.

### *B. subtilis* (and Its NusG) Is Not at All Like *E. coli*

The universal conservation of the NusG structure and its binding site on the RNAP, as well as perceived common principles of gene expression control in bacteria, justified using the *E. coli* NusG as a paradigm. However, early and recent data suggest that, beyond occupying the same site on RNAP, even housekeeping NusGs, which are encoded within the conserved genomic locus, *secE*-*nusG*-*rplK*-*rplA* in evolutionary distant bacterial phyla (Wang B. et al., 2020), have relatively few common features. Comparison of NusG proteins from *E. coli* and *B. subtilis*, the best studied Gram-negative and Gram-positive model bacteria that grow very similarly in the lab, illustrates these differences.

In wild-type *E. coli*, *nusG* and *rho* genes are essential; their deletions can be obtained only in specially engineered strains (Leela et al., 2013) and confer significant growth defects. In contrast, neither gene is essential in *B. subtilis* (Ingham et al., 1999), in which Rho has limited effects on gene regulation (Nicolas et al., 2012), early stop codons do not induce polarity (Johnson et al., 2020), and most transcription termination is induced by hairpin signals (Mondal et al., 2016; Johnson et al., 2020). In contrast to *E. coli*, where NusG aids Rho in termination of *rut*-less RNAs (Lawson and Berger, 2019), Rho-dependent termination in *B. subtilis* is strongly linked to *cis*-encoded C-rich RNA elements (Johnson et al., 2020). Together, these results suggest that NusG is not involved in gene expression control by Rho in *B. subtilis* (and perhaps other related bacteria) and raise a possibility that an alternative mechanism of transcription noise silencing operates in these species.

Another key function of *E. coli* NusG is bridging the RNAP and the ribosome (Figure 7) to mediate transcription-translation coupling, which is thought to occur in all single-compartment cells (see above). In addition to preventing Rho-dependent termination, which may be irrelevant in *B. subtilis*, the coupled ribosome inhibits RNAP backtracking (Proshkin et al., 2010; Stevenson-Jones et al., 2020) and could disfavor the formation of deleterious R-loops (Gowrishankar et al., 2013). The pioneer round of translation may also prime the RNA for subsequent rounds of translation. Strikingly, a recent report demonstrates that transcription and translation are uncoupled in *B. subtilis* (Johnson et al., 2020), where RNAP moves along the template about twice as fast as the ribosome does. While in *E. coli* the coupled ribosome inhibits both intrinsic and Rho-dependent termination, termination in *B. subtilis* is unaffected by translation. The loss of coupling has a profound effect on operon structure: more than 70% of *B. subtilis* intrinsic terminators are positioned just downstream of the stop codon (Johnson et al., 2020), where they would be rendered inefficient by the trailing ribosome in *E. coli* (Roland et al., 1988). These findings are consistent with *in vitro* comparative analysis of *B. subtilis* and *E. coli* RNAP, which shows that *B. subtilis* enzyme transcribes faster and pauses less (Artsimovitch et al., 2000). In contrast, their ribosomes move at similar rates and are unable to catch up with the run-away *B. subtilis* RNAP (Johnson et al., 2020); even if *B. subtilis* NusG binds to the RNAP and the ribosome, it cannot bridge this gap.

In *E. coli*, RNAP pauses frequently and NusG facilitates RNA synthesis (Herbert et al., 2010). By contrast, *B. subtilis* RNAP rarely pauses and NusG stimulates pausing *in vitro* and *in vivo* (Yakhnin et al., 2020a). Unlike *E. coli* NusG, which is positioned next to the non-template DNA strand in the TEC but is not known to recognize any specific DNA elements (Kang et al., 2018), *B. subtilis* NusG specifically binds to T-rich DNA sequences and delays RNA chain elongation (Yakhnin et al., 2016). NusG-dependent RNAP pausing is required for regulation of several operons in *B. subtilis* (Yakhnin et al., 2020b); for example, NusG-dependent pausing in the *trp* and *rib* leader regions provides time for recruitment of an RNA-binding protein TRAP and for riboswitching by flavin mononucleotide, respectively. Sequence-specific pausing through non-template DNA contacts has been first shown for RfaH (Artsimovitch and Landick, 2002), which recognizes 12-nt *ops* elements in the *E. coli* genome (Belogurov et al., 2009); RfaH-induced RNAP delay is thought to facilitate the ribosome recruitment to the nascent RNA (see below) in a handful of leader regions. The *ops* sequence is a perfect match to the consensus pause sequence that induces pausing in *E. coli* (Larson et al., 2014; Vvedenskaya et al., 2014) but has additional recognition determinants for RfaH (Zuber et al., 2018).

By contrast, in *B. subtilis*, NusG recognizes a simpler consensus TTNTTT motif and stimulates pausing genome wide, favoring forward translocation of RNAP (Yakhnin et al., 2020a). Sequences that induce intrinsic, NusG-independent pausing of *B. subtilis* enzyme are also very different from the consensus pause elements documented in *E. coli*, and backtracking is not observed (Yakhnin et al., 2020a). Although the mechanism and regulation of pausing appear to be distinct, slowing RNAP is expected to be essential in both *B. subtilis* and *E. coli*. Pausing determines the overall rate of RNA chain synthesis, is an obligatory step in termination, and facilitates recruitment of regulatory factors (Kang et al., 2019). In both *E. coli* and *B. subtilis*, pausing has been implicated in attenuation control and co-transcriptional folding of riboswitches and catalytic RNAs (Landick et al., 1985; Pan et al., 1999; Perdrizet et al., 2012; Yakhnin et al., 2019), and contributes to coupling of transcription and translation in *E. coli* (McGary and Nudler, 2013). Pausing-defective *E. coli* RNAP variants do not support cell growth but can be rescued by small-molecule ligands that slow the RNAP down (Artsimovitch et al., 2003). In contrast to *E. coli* RNAP, which readily pauses at consensus sequences without the aid of accessory factors (Artsimovitch and Landick, 2000; Larson et al., 2014; Vvedenskaya et al., 2014), *B. subtilis* RNAP relies on NusG to slow it down (Yakhnin et al., 2016, 2020a). In this light, NusG can be viewed as a pause-promoting accessory subunit, a regulatory mechanism that could be widespread in bacteria (Yakhnin et al., 2020b). Indeed, *Thermus thermophilus* NusG reduces the RNA synthesis rate (Sevostyanova and Artsimovitch, 2010) and mycobacterial NusG promotes intrinsic termination (Czyz et al., 2014).

Is there any common function of NusG proteins? The conservation of the *boxA* and *boxB* RNA elements, all Nus factors, ribosomal proteins, and SuhB suggests that similar *rrn*-TACs may form in *B. subtilis*, a hypothesis supported by a report that *rrn* antitermination can be achieved in a heterologous *E. coli*/*B. subtilis* system (Arnvig et al., 2008). Observations that *B. subtilis* cells lacking NusG do not show defects in rRNA transcription argue that NusG is not required for rRNA synthesis (Yakhnin et al., 2020a). However, given that the principal role of the *E. coli rrn*-TACs appears to be in chaperoning of the nascent RNA (Huang et al., 2020), an analogous complex, with or without NusG, may be required to ensure the correct rRNA folding and processing in *B. subtilis*.

## A Tussle for RNAP

In addition to housekeeping NusG/Spt5 proteins present in all free-living cells, many genomes encode one or more NusG paralogs (Wang B. et al., 2020). While the primary sequences of these proteins are very diverse, the high conservation of residues that comprise the high-affinity RNAP binding site suggests that all of them bind to the TEC similarly. Indeed, *E. coli* NusG and RfaH, which are only 17% identical, make very similar contacts to that RNAP β’ subunit (Kang et al., 2018). However, in contrast to housekeeping NusG, which binds to RNAP and modulates transcription genome wide (Mooney et al., 2009a; Yakhnin et al., 2020a), these paralogs control expression of just a few target genes. Akin to alternative transcription initiation factors, these specialized NusGs (NusG^*SP*^) comprise a set of alternative transcription elongation factors that compete for the transcribing RNAP, an analogy further strengthened by their recruitment to the same site on RNAP (Sevostyanova et al., 2008).

**FIGURE 8:**
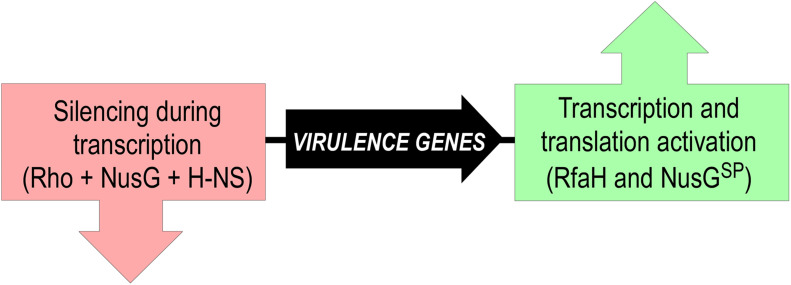
Silencing and counter-silencing of virulence genes by NusG-like proteins.

However, this analogy does not extend to functions and mechanisms of gene-specific recruitment. Every σ factor activates transcription of its cognate promoters by recruiting RNAP and facilitating DNA melting; just the promoter sequences differ. In a stark contrast, NusG^*SP*^ factors activate expression of genes that the housekeeping NusG silences (Figure 8). These genes can be a few in number, but critical for bacterial evolution and pathogenesis because they encode conjugation and virulence determinants (see below).

**FIGURE 9:**
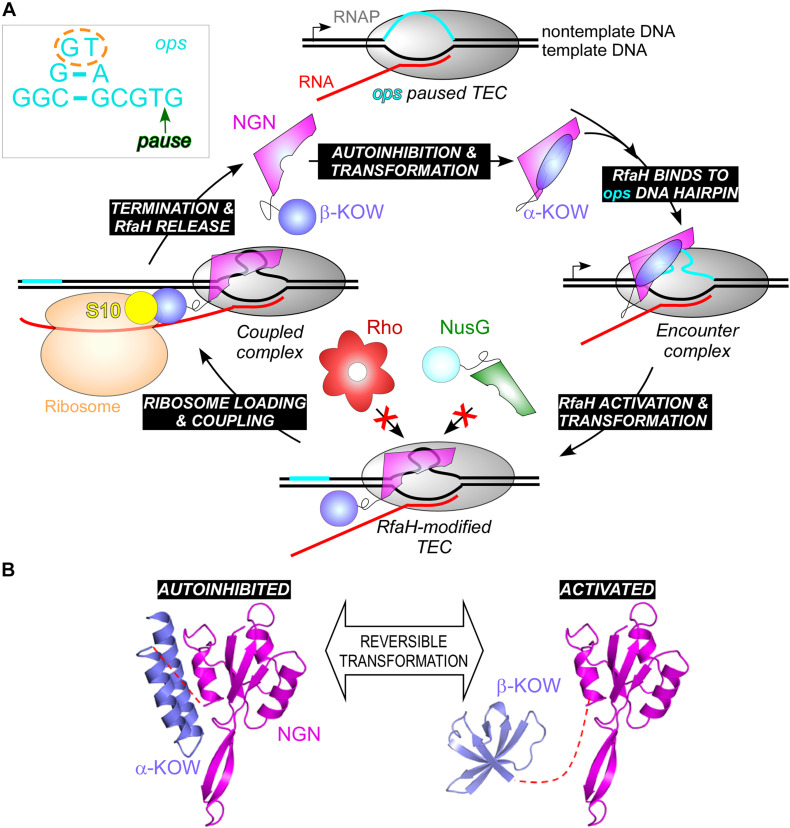
**(A)** A full cycle of RfaH; see text for details. The inset shows the *ops* DNA element, which forms a short hairpin on the TEC surface; *ops* bases that make most interactions with RfaH in the complex are circled; the pause position is indicated by an arrow. **(B)** RfaH domain dissociation and refolding. PDB IDs: autoinhibited RfaH, 5OND; activated RfaH, 6C6S.

Furthermore, while σ factors bind to specific DNA sequences in static promoter complexes, NusG homologs are recruited to a moving RNAP. The available data suggest that these proteins use different recruitment mechanisms, only in some cases relying on specific protein-DNA interactions. Housekeeping NusGs are abundant proteins that can bind the TEC by chance, irrespective of the transcribed sequence; indeed, specific interactions would slow RNAP down, a regulatory feature used in *B. subtilis* (Yakhnin et al., 2020a) but not in *E. coli*, in which NusG is sequence blind. By contrast, the best characterized NusG^*SP*^, *E. coli* RfaH, uses a very complex mechanism to ensure efficient and selective recruitment to its targets (Zuber et al., 2018). RfaH is recruited to the TEC at operon polarity suppressor (*ops*; Figure 9A) sites (16 in *E. coli* MG1655 genome) which are present in leader regions of several operons silenced by NusG and Rho (Artsimovitch and Knauer, 2019). The *ops* element is a composite regulatory signal: it induces RNAP pausing and backtracking (Artsimovitch and Landick, 2000) and is directly recognized by RfaH (Artsimovitch and Landick, 2002). Pausing at *ops* is essential for RfaH recruitment (Zuber et al., 2018): it (*i*) provides additional time for RfaH, which is present in few copies/cell, to find its target; and (*ii*) presents the *ops* bases in a small hairpin, with a conserved T residue flipped out for specific recognition by RfaH (Kang et al., 2018; Zuber et al., 2018). This is a one-time opportunity because, once the RNAP moves past *ops*, the recruitment window is closed; thus, RfaH must bind to RNAP at *ops* and stay bound until the end of RNA synthesis. To fend off 100-fold more abundant NusG (Schmidt et al., 2016), RfaH binds RNAP much tighter (Kang et al., 2018), essentially becoming an RNAP subunit for one round of RNA synthesis. RfaH maintains the ability to trigger pausing at a downstream (engineered) *ops* site while traveling with RNAP but reduces pausing at any other sequence (Belogurov et al., 2009).

If RfaH binds to RNAP very tightly during elongation, why does it need the *ops* signal in the first place? Unlike NusG, in which the RNAP-binding site on the NGN domain is exposed, this site is blocked by the KOW domain in free RfaH (Figure 9). Also unlike NusG, in which the KOW domain is in a β-barrel state (β-KOW; Figure 2), in this “autoinhibited” RfaH the KOW domain is folded as an α-helical hairpin (α-KOW; Figure 9B). To bind RNAP, RfaH must be “activated” by domain dissociation, which happens only in the presence of a complete *ops*-paused TEC (Zuber et al., 2019). The details of this process remain elusive, but the current model suggests that the NGN domain recognizes the *ops* hairpin *via* its exposed DNA-binding residues, forming a transient encounter complex and triggering the KOW dissociation (Artsimovitch and Knauer, 2019). It is possible that autoinhibition may be a common feature of NusG homologs. While in *E. coli* NusG the NGN and KOW domains move freely (Burmann et al., 2011), in NusG from a hyperthermophilic bacterium *Thermotoga maritima*, the two domains interact, masking the binding sites for RNAP, NusE, and Rho (Drögemüller et al., 2013). Domain dissociation enables *T. maritima* NusG-KOW binding to Rho and NusE, and these contacts may be stabilized by the NGN-RNAP contacts (Drögemüller et al., 2017).

RfaH recruitment relies on the multi-functional DNA element and elaborate structural rearrangements of the protein domains. Binding to a specific DNA element enables RfaH to control several operons scattered on the chromosome. But how is a wannabe NusG^*SP*^, which has just surfaced following gene duplication, targeted to a specific locus in the presence of overwhelming numbers of NusG molecules? An “ancestral” mechanism, in which NusG^*SP*^ binds to the transcribing RNAP *in cis* has been proposed to explain this conundrum (Belogurov et al., 2009). This model is supported by bioinformatics analyses which reveal that the residues that mediate DNA contacts in RfaH arose late in evolution and that many NusG^*SP*^ are encoded within long xenogeneic operons, in contrast to the standalone *rfaH* gene (Wang B. et al., 2020). However, observations that some of these *cis*-encoded regulators act *in trans* (Chatzidaki-Livanis et al., 2010) suggest that NusG^*SP*^ recruitment strategies are multifaceted.

## Structural Transformation of RfaH

RfaH activation is not limited to the domain dissociation needed to expose the RNAP-binding site: the released α-KOW undergoes a dramatic transformation into a NusG-like β-KOW (Figure 9B) and binds to S10 similarly to NusG KOW (Burmann et al., 2012). The residues that make contacts with S10 are not available in the α-KOW domain, thus the free RfaH is autoinhibited with respect to both RNAP and ribosome binding, allowing RfaH to achieve high target specificity (Shi et al., 2017). The activated state persists until the TEC dissociates at a terminator and RfaH is released; the KOW then refolds into the α-helical hairpin and re-establishes contacts with the NGN, restoring autoinhibition (Figure 9A).

Interconversion between the alternative RfaH-KOW states is principally controlled by interdomain contacts: the KOW (re)folds into a β-barrel when expressed alone, separated from the NGN domain upon proteolytic cleavage of the linker, or as a result of interface-destabilizing substitutions (Burmann et al., 2012; Tomar et al., 2013; Shi et al., 2017). Deuteron incorporation reveals that the tip of the C-terminal α-hairpin is stably folded in the autoinhibited state, whereas the rest of the KOW is highly flexible, and its flexibility only decreases in the β-folded state (Galaz-Davison et al., 2020). The mechanism underlying this dramatic fold switch has been also pursued by computational approaches (Gc et al., 2014, 2015; Balasco et al., 2015; Ramírez-Sarmiento et al., 2015; Xiong and Liu, 2015; Xun et al., 2016). Although the β-barrel is a preferred state of the isolated RfaH-KOW, its free energy is only slightly lower than that of the α-helical conformation. The separation of the two alternative states is dependent on large energy barriers resulting from the main chain hydrogen bonds of the α-helical hairpin. An all-atom Monte Carlo simulations study suggests a possibility that the encounter complex between the autoinhibited RfaH and the *ops*-TEC is characterized by net attractive interactions with the NGN and net repulsive interactions with the KOW. The resulting opposing forces on the two domains, in combination with the peculiar mechanical rigidity profile of the autoinhibited RfaH, might help trigger domain separation (Seifi et al., 2020). The α→β rearrangement essentially depends on an unstructured state: upon dissolution of the α-helical hairpin, the KOW assumes a disordered state and then follows a step-wise assembly into the final five-stranded β-barrel (Bernhardt and Hansmann, 2018; Joseph et al., 2019).

Among NusG homologs, *E. coli* RfaH is the only known transformer protein. However, it is possible that other KOW domains are capable of transformation. In particular, an amazingly broad repertoire of known cellular targets of eukaryotic NusG homologs (Decker, 2020) could be due to metamorphic behavior of their KOWs.

## RfaH as a Translation Factor

**FIGURE 10:**
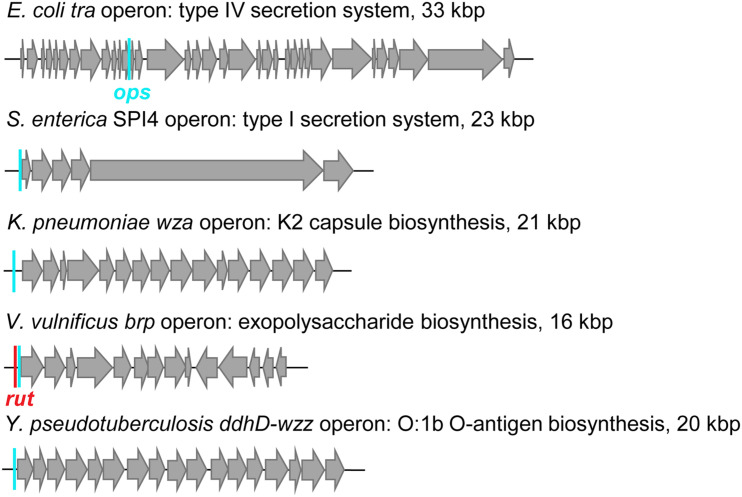
Examples of RfaH-controlled operons. Positions of *ops* sites (cyan bars) and a *rut* site (red bar) are indicated.

RfaH-controlled genes encode toxins, adhesins, LPS and capsule biosynthesis enzymes, type IV secretion apparatus, etc. located in long horizontally acquired operons (Figure 10), which are silenced by Rho. RfaH abolishes Rho-dependent termination (Sevostyanova et al., 2011) and the ability to bind Rho appears to be lost early in RfaH evolution (Wang B. et al., 2020). RfaH elicits dramatic, 50 + fold activation of gene expression *in vivo*, an effect that was initially assumed to be mediated by its direct antitermination effects on RNAP (Artsimovitch and Landick, 2002). Surprisingly, RNAP modification by RfaH makes only a minor contribution in the cell (Sevostyanova et al., 2011). Instead, RfaH inhibits Rho-dependent termination by outcompeting NusG and activating translation.

RfaH-controlled genes lack Shine-Dalgarno elements, which recruit the ribosome through RNA base-pairing with the 16S rRNA (Rodnina, 2018) and have many rare codons, limiting their translation and making them easy targets for Rho. Observations that the transformed β-KOW directly binds S10 (Burmann et al., 2012) prompted a hypothesis that RfaH recruits the ribosome via β-KOW/S10 contacts and then couples transcription to translation during elongation. In support of this model, expression of SD-less reporters is completely dependent on RfaH, and substitutions of residues that interact with S10 abolish expression (Burmann et al., 2012). In addition to the ribosome recruitment, by bridging the RNAP and the ribosome during elongation, RfaH may prevent uncoupling at rare codons; the ribosome stalling exposes mRNA to Rho (Elgamal et al., 2016). RfaH may be particularly important during synthesis of excessively long proteins such as *Salmonella* pathogenicity island IV giant 600 kDa adhesin (Figure 9B), which requires RfaH for expression (Main-Hester et al., 2008). Remarkably, the *ops*-RfaH module supports efficient expression of an SD-less reporter *in vivo*, ∼20% relative to that driven by a perfect SD element (Burmann et al., 2012).

Although RfaH and NusG make similar contacts to S10 (Burmann et al., 2012), their effects on translation are expected to be different. NusG binds to the RNAP transiently (Kang et al., 2018) and late in the operon, well after the first ORF (Mooney et al., 2009a). In contrast, RfaH binds to RNAP upstream of the first ORF and remains stably associated with the EC until termination (Belogurov et al., 2009). It is possible that RfaH recruits the ribosome to the *ops*-paused RNAP and promotes ribosome scanning for a downstream initiation codon. Future studies will reveal the details of translation activation by RfaH, but the available data suggest that this universally conserved transcription antiterminator may be acting primarily as an RNAP-tethered translation initiation/elongation factor and may employ the first protein-mediated ribosome recruitment mechanism outside of viruses.

## Diversity of the NusG Family

**FIGURE 11:**
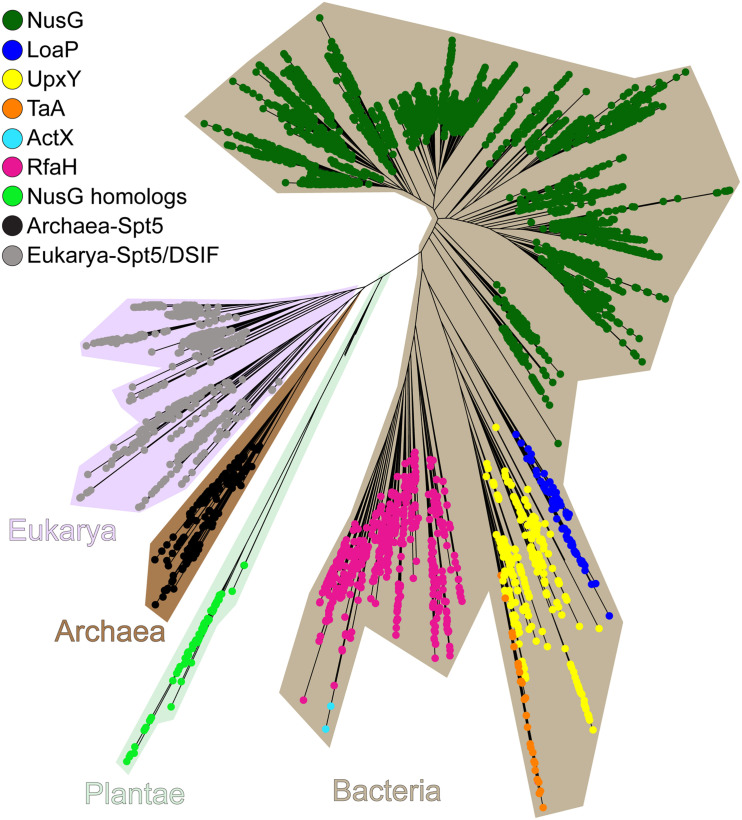
The maximum-likelihood phylogenetic tree of NusG-like proteins. Plantae is an artificial group used solely for brevity.

Specialized NusG paralogs (Figure 11) are evolving in very different ecological niches but may have similar functions—to promote expression of long or silenced operons. Functional data implicate several NusG^*SP*^ in transcription antitermination of very long gene clusters, whereas for others this function is inferred from their genomic associations. *Bacillus amyloliquefaciens* LoaP inhibits termination in two operons producing antibiotics difficidin and macrolactin (Goodson et al., 2017). Differently from RfaH, which is rather inefficient against intrinsic terminators (Artsimovitch and Landick, 2002; Carter et al., 2004), LoaP promotes readthrough of the hairpin termination signals (Goodson et al., 2017). Polyketide antibiotic TA made by *Myxococcus xanthus* inhibits bacterial cell wall synthesis and is produced by a 40 kb operon which is activated by NusG^*SP*^ called TaA (Paitan et al., 1999) by an unknown mechanism. Human gut bacterium *Bacteroides fragilis* synthesizes eight capsular polysaccharides from separate operons, which are activated by UpxY family of NusG^*SP*^. UpxY proteins prevent premature transcriptional termination within the 5′ leaders upstream from the *upxY* gene (Chatzidaki-Livanis et al., 2009).

While functional data are available for just a few NusG^*SP*^, recent bioinformatics analysis suggests that these proteins fall into eight different clusters, which differ in their primary sequence signatures as well as regulatory contexts. Some NusG^*SP*^, such as RfaH, form one group and are encoded by single cistrons, whereas others (e.g., *loaP*, *taA*, and *upxY*) are adjacent to their target operons (Wang B. et al., 2020). ActX, which is closely related to RfaH (Figure 11), is encoded within pilus biosynthesis operons on antibiotic-resistant plasmids in *E. coli* and *Klebsiella pneumoniae* (Núñez et al., 1997), but its regulatory function remains unknown. Analysis of genomic contexts can be instrumental in predicting functional associations (Moreno-Hagelsieb and Santoyo, 2015). Gene neighbors of NusG^*SP*^ (except for RfaH-like stand-alone genes) are enriched in genes involved in cell envelope biogenesis, with glycosyltransferases, nucleoside-diphosphate-sugar epimerases, and exopolysaccharide biosynthesis enzymes being the most common (Wang B. et al., 2020). However, notable differences exist among distinct clusters; for example, some NusG^*SP*^ are adjacent to Tat protein secretion system, others are encoded near undecaprenyl pyrophosphate synthase and H-NS genes. A group of regulators from *Shewanella* are encoded within putative exopolysaccharide operons, an arrangement resembling *B. fragilis* operons controlled by UpxY proteins (Chatzidaki-Livanis et al., 2010). Future studies will be required to determine functional significance of these associations.

Extensive duplications, sub-functionalization, and horizontal transfer underpin the evolution of NusG paralogs. One NusG copy has gradually evolved into RfaH, starting from an “early” loss of binding to Rho terminator while tightening contacts to RNAP and culminating with the “late” acquisition of residues that interact with the *ops* DNA element and confer autoinhibition (Wang B. et al., 2020). While in most NusG homologs these changes do not alter the core domain structure, some factors acquired additional domains thought to promote adaptation to their unique niches. For example, in *T. maritima* NusG, an extra domain DII supports NusG recruitment to the TEC and stabilizes the NusG:RNAP complex, a necessary adaptation to high temperatures in the *T. maritima* natural habitat (Drögemüller et al., 2017).

In addition to Spt5, NusG homologs are also encoded in the genomes of all major land plant and algal lineages except for some green algal species (Wang B. et al., 2020). These bacterial regulators have recognizable chloroplast-localization signals and are presumably retained to assist the bacterial-type RNAPs that mediate chloroplast transcription. A NusG homolog *of Arabidopsis thaliana* has been identified as a component of the active transcriptional machinery in chloroplasts (Pfalz et al., 2006), and a Rho ortholog has been shown to terminate transcription by plastid-encoded RNAP (Yang et al., 2020).

## NusG Paralogs and Virulence

Extensive functional studies have established RfaH as the paradigm for the regulation of transcription elongation, translation initiation, and protein folding. However, RfaH is also a key virulence factor. RfaH activates the expression of capsule, cell wall, toxins, adhesins, and pilus biosynthesis operons (Figure 9B), which are important for virulence and conjugal transfer in several Gram-negative pathogens including *E. coli*, *K. pneumoniae*, *Vibrio vulnificus*, *Salmonella enterica*, *Yersinia pseudotuberculosis*, and *Yersinia pestis* (Kong et al., 2011; Bachman et al., 2015; Garrett et al., 2016; Hoffman et al., 2017). RfaH effects on gene expression are very large (50+ fold); consequently, the loss of *rfaH* leads to dramatic defects in virulence, e.g., 10^4^ decrease in *K. pneumoniae* survival in the lung (Bachman et al., 2015).

The first protein secretion process discovered in bacteria was the hemolysin A (HlyA) type 1 secretion system (T1SS), which is found in uropathogenic *E. coli* strains (Thomas et al., 2014). HlyA is a 107 kDa protein that induces hemolysis by creating pores in the erythrocyte membrane (Skals et al., 2009). RfaH, a.k.a. HlyT, has been identified genetically as an activator of the *hly* operon (Thomas et al., 2014). Inactivation of *rfaH* dramatically decreases virulence of uropathogenic *E. coli* strain in a murine model of urinary tract infection (Nagy et al., 2002). The capability to colonize the intestinal tract by efficiently competing with the commensal microbiota has been considered as a multifactorial virulence property. RfaH also plays a role in the infectious process during colonization of the intestinal tract: *rfaH* mutants are susceptible to bile salts and show reduced gut colonization capacity (Nagy et al., 2005).

Antibiotic-resistant *K. pneumoniae* is an urgent public health threat and a leading cause of pneumonia in hospitalized patients (David et al., 2019). Functional genomic profiling of four diverse serum-resistant *K. pneumoniae* strains reveals that the deletion of *rfaH* dramatically reduces resistance to serum complement system in all strains (Short et al., 2020). *Vibrio vulnificus* is another opportunistic human pathogen responsible for the majority of seafood-associated deaths worldwide, and antibiotic resistance has developed (Heng et al., 2017). Loss of *rfaH* also makes *V. vulnificus* highly sensitive to human serum (Garrett et al., 2016). Expression of the *brp* exopolysaccharide operon mediates surface adherence of *V. vulnificus*, and the presence of *ops* and *rut* sites in the leader region suggests RfaH-dependent antitermination (Chodur and Rowe-Magnus, 2018). *S. enterica* serovar Typhimurium is a primary enteric pathogen infecting both humans and animals and a major cause of diarrheal diseases, with antibiotic resistance on the rise (Fàbrega and Vila, 2013; Knodler and Elfenbein, 2019). *Salmonella* harbors five pathogenicity islands (SPI) required for infection in vertebrate hosts. Among them, SPI4 plays a role in the initial interaction with the intestinal epithelium and possibly contributes to long-term persistence (Gerlach et al., 2007). *S. enterica* RfaH is required for the expression of SPI4, which encodes a T1SS and its adhesin substrate (Main-Hester et al., 2008), as well as the expression of secreted and surface-associated polysaccharides (Lindberg and Hellerqvist, 1980; Bailey et al., 1997). Mutants of *S. enterica* serovar Typhimurium lacking *rfaH* are efficient as vaccines against salmonellosis and induce strong serum immune responses (Nagy et al., 2006; Liu et al., 2016). Given their association with capsular and TSS operons (Wang B. et al., 2020), other NusG paralogs likely play important roles during pathogenesis.

Antibiotic resistance determinants are frequently encoded on conjugative plasmids and can be rapidly transferred between bacteria (Wang et al., 2017). RfaH activates the F plasmid conjugation operon (Beutin and Achtman, 1979) and RfaH homologs are encoded on some clinical resistant plasmids (Wang B. et al., 2020), suggesting that they may contribute to plasmid transfer. A recent study showed that deletions of seven genes, including *rfaH*, prevented cefotaxime-induced up-regulation of *traF* and decreased the conjugative transfer of the resistance plasmid (Liu et al., 2019).

RfaH proteins from *Vibrio*, *Yersinia*, *Salmonella*, and *Klebsiella* bind to the *E. coli* TEC *in vitro* and complement the *E. coli rfaH* gene deletion (Carter et al., 2004). Small molecule inhibitors that block recruitment of *E. coli* and *K. pneumoniae* RfaH to RNAP (Svetlov et al., 2018) may have a potential to inhibit virulence and the spread of antibiotic resistance.

## Concluding Remarks

NusG homologs comprise the only universally conserved family of transcription factors, which includes housekeeping regulators and their specialized paralogs (Figure 11). Despite highly similar core domain architectures and interactions with RNAP, NusG-like proteins exert amazingly diverse, and frequently opposite, effects on gene expression. Bacterial NusG homologs can inhibit or stimulate transcription termination, accelerate RNA synthesis by suppressing RNAP backtracking or slow transcription down by halting RNAP at specific sequences, bridge the RNAP to the ribosome during translation elongation or recruit the ribosome to mRNAs that lack canonical ribosome binding sites, and likely perform other functions that remain to be discovered.

This regulatory plasticity depends on dynamic interactions of the NGN and KOW domains with each other, RNAP, single and double-stranded nucleic acids, and many auxiliary cellular proteins. While bound to the TEC through contacts mediated by highly conserved residues within RNAP and NGN, NusG homologs employ divergent residues in their NGN and KOW domains to enact a range of responses demanded by specific cellular circumstances. Some NusG paralogs augment their regulatory prowess by undergoing an unprecedented and reversible refolding of an entire KOW domain, during which the protein turns inside out. The presence of NusG in all free-living organisms, sometimes in several copies, confirms its unique place in gene expression control, from LUCA to present life forms.

## Author Contributions

BW prepared all original figures and wrote the first draft. IA revised and expanded the manuscript. Both authors prepared figures and edited the draft while preparing a revised manuscript.

## Conflict of Interest

The authors declare that the research was conducted in the absence of any commercial or financial relationships that could be construed as a potential conflict of interest.
